# Dr. Macnish

**Published:** 1837-04

**Authors:** 


DR. MACNISH.

At Glasgow, on the 26th December, 1836, in the thirty-fifth year of his age. Dr. Macnish was a man of great talent, and the author of several works of much interest and value, the principal of which are "The Anatomy of Drunkenness" and "The Philosophy of Sleep."

